# Comparative Analysis of the Global Transcriptome of *Anopheles funestus* from Mali, West Africa

**DOI:** 10.1371/journal.pone.0007976

**Published:** 2009-11-19

**Authors:** Andrew C. Serazin, Ali N. Dana, Maureen E. Hillenmeyer, Neil F. Lobo, Mamadou B. Coulibaly, Michael B. Willard, Brent W. Harker, Igor V. Sharakhov, Frank H. Collins, Jose M. C. Ribeiro, Nora J. Besansky

**Affiliations:** 1 Eck Institute for Global Health, Department of Biological Sciences, University of Notre Dame, Notre Dame, Indiana, United States of America; 2 Malaria Research and Training Center, Bamako, Mali; 3 Laboratory of Malaria and Vector Research, National Institute of Allergy and Infectious Diseases, National Institutes of Health, Bethesda, Maryland, United States of America; Institut Pasteur, France

## Abstract

**Background:**

*Anopheles funestus* is a principal vector of malaria across much of tropical Africa and is considered one of the most efficient of its kind, yet studies of this species have lagged behind those of its broadly sympatric congener, *An. gambiae*. In aid of future genomic sequencing of *An. funestus*, we explored the whole body transcriptome, derived from mixed stage progeny of wild-caught females from Mali, West Africa.

**Principal Findings:**

Here we report the functional annotation and comparative genomics of 2,005 expressed sequence tags (ESTs) from *An. funestus*, which were assembled with a previous EST set from adult female salivary glands from the same mosquito. The assembled ESTs provided for a nonredundant catalog of 1,035 transcripts excluding mitochondrial sequences.

**Conclusions/Significance:**

Comparison of the *An. funestus* and *An. gambiae* transcriptomes using computational and macroarray approaches revealed a high degree of sequence identity despite an estimated 20–80 MY divergence time between lineages. A phylogenetically broader comparative genomic analysis indicated that the most rapidly evolving proteins– those involved in immunity, hematophagy, formation of extracellular structures, and hypothetical conserved proteins– are those that probably play important roles in how mosquitoes adapt to their nutritional and external environments, and therefore could be of greatest interest in disease control.

## Introduction

About 90% of malaria deaths worldwide occur in Africa [1]. This disproportionate burden is due to the intensity of *Plasmodium falciparum* transmission by three widespread and efficient mosquito vectors: *Anopheles gambiae*, its closely related sibling species *An. arabiensis*, and a more distant relative, *An. funestus* [2]. *Anopheles gambiae* and *An. funestus* share particularly anthropophilic tendencies that contribute strongly to their vectorial capacity [3]. Nevertheless, ecological and behavioral differences exist that have important epidemiological consequences. Whereas *An. gambiae* typically breeds in small temporary rain-dependent pools and puddles, *An. funestus* exploits large permanent or semi-permanent bodies of water containing emergent vegetation. It attains maximal abundance in the dry season after densities of *An. gambiae* and *An. arabiensis* have declined, thereby extending the period of malaria transmission [2]. To be successful, malaria control strategies aimed at the mosquito should consider the unique biology of *An. funestus* and other relatively neglected vector species [4].

Despite its importance in malaria transmission, few studies have been directed at genetic analysis of *An. funestus* until recently. Early efforts were hampered by inefficient or missing tools: lack of laboratory colonies, cumbersome methods for species identification, and the absence of molecular markers, genetic maps, and other resources. Important advances in the past few years have begun to address these deficiencies [4]–[6], though more attention is still needed to translate these advances into tools for control. *Anopheles funestus* is significant in its own right as a target of public health intervention, justifying further investment. Beyond that, comparative genomics involving *An. funestus* and additional anopheline genomes is further motivation, as it will provide both context for functional annotation of the reference *An. gambiae* genome, and a platform for the genetic analysis of traits associated with successful human malaria vectors. As of 2009, *An. gambiae* was the only sequenced representative of Anophelinae, the mosquito subfamily that contains all known human malaria vectors. The only other completely sequenced mosquito genomes are classified in a different subfamily, Culicinae. These species, *Aedes aegypti* and *Culex quinquefasciatus*, are major vectors of Yellow Fever, Dengue and West Nile viruses but are refractory to infection by human malaria parasites and very distantly related to anophelines, having diverged from a common ancestor ∼145–200 million years ago [7].

In aid of future genomic sequencing and SNP discovery, we explored the *An. funestus* whole body transcriptome, derived from mixed stage progeny of wild-caught females from Mali, West Africa. Here we report the functional annotation and comparative genomics of 2,005 expressed sequence tags (ESTs) from *An. funestus*, which were assembled with a previous EST set from adult female salivary glands from the same mosquito [8]. The assembled ESTs provided for a nonredundant catalog of 1,035 transcripts when mitochondrial sequences were excluded.

## Materials and Methods

### cDNA Library Construction

Blood-engorged adult female *An. funestus* mosquitoes were collected inside houses from Niono, Mali. The progeny of these females, approximately 50 individuals including larvae, pupae, and adult males and females, were used to construct a cDNA library representative of multiple developmental stages. From total RNA isolated with Trizol (Molecular Research Center, Inc), mRNA was extracted using the PolyATract mRNA Isolation System (Promega) and converted to cDNA based on the SMART cDNA library construction kit (Clontech, Palo Alto, CA). First-strand cDNA synthesis was carried out at 42°C for 1 h using Superscript II Reverse Transcriptase (Life Science Technology, MD) with a modified oligo (dT) primer, CDS III (3′) containing a *Sfi* IB restriction site, and an additional primer (SMART III) that adds an *Sfi* IA restriction site at the 5′ end of the cDNA for directional cloning. Second-strand synthesis was conducted in the presence of both primers using Advantage 2 Polymerase Mix (Clontech), under the following PCR conditions: 95°C for 20 s, followed by 22 cycles of 95°C for 5 s and 68°C for 6 min, concluding at 72°C for 10 min. Following proteinase K digestion and phenol:chloroform extraction, the amplified cDNAs were digested with *Sfi* I at 50°C for 2 h and size fractionated using CHROMA SPIN-400 columns (Clontech). Fractions containing cDNAs longer than 500 bp, as judged by 1% agarose gel electrophoresis, were pooled, ethanol precipitated, and ligated into λTripIEx2 (Clontech). Ligation mixtures were packaged using Gigapack III Gold Packaging Extract (Stratagene, La Jolla, CA) and incubated with log phase *E. coli* XL1-Blue cells (Stratagene). Unamplified library titer was estimated at 1.4×10^6^ independent clones.

### cDNA library sequencing

A total of 3264 recombinant plaques were plugged and transferred into individual wells of a 96-well plate containing 100 µL of 2% chloroform/SM buffer (0.1 M NaCl, 0.01 M MgSO_4_, 0.05 M Tris-HCl pH 7.5, 0.01% gelatin). Following overnight elution, cDNA inserts were amplified in 25 µL PCR reactions containing 0.4 µL of phage suspension, 0.03 pmol each of 3′ and 5′ LD Amplimer primers (Life Technologies), 1X Taq Polymerase Buffer (Invitrogen), 3 mM MgCl_2_, 1 mM of each dNTP, and 0.2 U Taq Polymerase (Invitrogen). Amplification reactions were performed in 96-well plates on a Perkin-Elmer 9700 Thermocycler (Applied Biosystems, Foster City, CA) with an initial denaturation at 95°C for 5 min, followed by 25 cycles of 94°C for 30 s and 70°C for 2 min, and a final 68°C for 3 min. Eight random samples from each 96-well plate were analyzed on a 1% agarose gel to confirm the absence of contamination and visible primer-dimer, indicating that PCR products could be sequenced without further purification.

Sequence was determined from 0.7 µL PCR product and 7.4 pmol of the 5′ LD Amplimer primer using the ABI PRISM Big Dye Terminator 3.0 Cycle Sequencing kit (Applied Biosystems) and an ABI 3700 Sequencer. ABI trace files were acquired for 3020 of the 3264 sequenced clones. Sequences have been deposited into the dBEST database (GenBank) under accession numbers CD576727-CD578395 and CD664201-CD664267.

### EST processing, clustering and bioinformatics

ESTs were trimmed to omit primer and vector sequences, poly(A) regions longer than 15 bp, and bases with a Phred quality score <16 using DNAStar SeqMan II (v5.03) software (DNAstar, CA). Any read sharing five 16-mer segments with the *Anopheles* mitochondrial or *E. coli* genome was counted as a contaminant. The remaining 1037 sequences were assembled into clusters (≥two ESTs) and singletons (one EST) based on the following criteria: sequence match size of 20 bp, minimum match percentage of 85%, 0.00 gap opening penalty, 0.7 gap length penalty, and minimum sequence length of 70 bp. Sequences were then manually examined to ensure a minimum length of 100 bp. In addition, these same sequences were re-clustered together with ESTs previously reported by Calvo et al. [8] that were derived from the salivary gland transcriptome of adult female *An. funestus,* using clustering procedures described by Valenzuela et al. [9].

Functional annotation of ESTs was based on BLASTX [10] with the filter for low complexity set to False (-F F) and word size = 2 (-W 2), using cluster consensus and singleton sequences as queries against the nonredundant (NR) and Gene Ontology [GO; 11] databases and various organism proteomes downloaded from NCBI (*Saccharomyces cerevisiae*, *Caenorhabditis elegans*, *Arabidopsis thaliana*), Flybase (*Drosophila melanogaster*), and Vectorbase (*An. gambiae, Ae. aegypti, Cx. quinquefasciatus*). Significant sequence similarity was defined as an expect (E) value <1×10^−4^. Functional annotation also considered conserved protein domains identified through searches of Pfam [12], SMART [13], Kog [14], and CDD [15] databases using RPS-BLAST [10]. Based on the combined results of BLASTX and protein domain searches, transcripts were presumptively assigned to one of 23 broad functional categories (see Table 1). Coding sequences (CDS) were deduced from the assembled ESTs by a semi manual process using a program (Assembler_Joiner) written by JMCR, where in-frame coding nucleotide sequences were extracted and frame shifts were corrected.

**Table 1:** Functional classification of *An. funestus* EST clusters.

Class	No. (%) of Clusters	No. (%) of ESTs	ESTs/Cluster
Salivary	54 (5.2)	484 (24.1)	9.0
Nuclear regulation	7 (0.7)	10 (0.5)	1.4
Transcription factor	5 (0.5)	7 (0.3)	1.4
Transcription machinery	10 (1.0)	17 (0.8)	1.7
Protein synthesis machinery	68 (6.6)	118 (5.9)	1.7
Protein export machinery	19 (1.8)	29 (1.4)	1.5
Protein modification machinery	43 (4.2)	71 (3.5)	1.7
Proteasome machinery	11 (1.1)	16 (0.8)	1.5
Transporters/storage	17 (1.6)	33 (1.6)	1.9
Oxidant metabolism/detoxification	11 (1.1)	16 (0.8)	1.5
Metabolism, carbohydrate	15 (1.4)	22 (1.1)	1.5
Metabolism, nucleotide	6 (0.6)	8 (0.4)	1.3
Metabolism, amino acid	6 (0.6)	8 (0.4)	1.3
Metabolism, lipid	8 (0.8)	10 (0.5)	1.3
Metabolism, intermediate	3 (0.3)	5 (0.2)	1.7
Signal transduction	31 (3.0)	58 (2.9)	1.9
Extracellular matrix/cell adhesion	50 (4.8)	132 (6.6)	2.6
Cytoskeletal	35 (3.4)	119 (5.9)	3.4
Transposable element	4 (0.4)	4 (0.2)	1.0
Metabolism, energy	66 (6.4)	176 (8.8)	2.7
Unknown	414 (40.0)	450 (22.4)	1.1
Unknown, conserved	132 (12.8)	185 (9.2)	1.4
Immunity	20 (1.9)	27 (1.3)	1.4
Total	1035	2005	

More detailed comparative genomic analysis was based on a subset of CDS that exceeded a quality cut-off of 60 based on the raw self-BLAST score, or “reference” score. This approach, rather than a threshold based on amino acid length, better accommodated small peptide classes (*e.g.*, those involved in defense) and incomplete proteins. To derive self-BLAST reference scores, the *An. funestus* conceptual proteins were gathered into a database that was searched by BLASTP with each component protein, with the filter for low complexity set to False (-F F), word size = 2 (-W 2) and using the default compositional matrix adjustment ON. The BLAST raw score for each *An. funestus* protein against itself comprised the reference score; proteins scoring ≥60 were retained for further analysis. Similarity to proteins in other databases adopted the BLAST Score Ratio approach [16]. In this approach, the best raw BLAST score resulting from a comparison between an *An. funestus* protein query and another proteome is divided by the reference *An. funestus* self-BLAST raw score, producing a normalized BLAST score. Accordingly, the normalized scores vary from 1 (a perfect match) to 0 (no match). Using normalized raw BLAST scores in this way overcomes several problems associated with the use of E-values, including (1) biases entailed in comparisons among different databases; (2) falsely high E-values assigned to low-complexity proteins such as mucins; and (3) falsely low E-values based on small regions of high similarity.

To identify orthologous gene pairs between *An. funestus* and *An. gambiae*, *An. funestus* ESTs were compared by TBLASTX to *An. gambiae* EST sequences available in dbEST (April 2003). ESTs matching with an E-value <1×10^−10^ were considered putative homologs. Each homolog of the gene pair was searched with TBLASTX against the *An. gambiae* genome. Identical top BLAST hits suggested the genes were putative orthologs; non-identical top BLAST hits suggested the possible presence of paralogs, which were excluded from the analysis. (The absence of an *An. funestus* whole genome assembly precluded the usual strategy of testing for reciprocal best BLAST hits.) Orthologous gene pairs were aligned by hand and corrected for reading frame shifts. If the aligned coding region was less than 50 amino acids in length, the genes were excluded from the analysis. Base composition (G+C content) at the third nucleotide position of codons was measured using the method of Wright [17], implemented in DnaSP 3.51 [18]. Codon usage bias was measured by ENC [17] and CBI [19], both implemented in DnaSP 3.51. ENC (the “effective number of codons” used in a gene) ranges from 20 codons (maximum bias) to 61 codons (no bias). CBI (codon bias index) measures deviation from equal use of synonymous codons and ranges from 0 (no bias) to 1 (maximum bias).

### Custom macroarray

Spotted probes consisted of cDNA inserts amplified as described in section 2.2, except in a reaction volume of 100 µL containing 1.0 µL eluted phage. A subset of amplified cDNA inserts (435 of 704) were checked by electrophoresis on a 1% agarose gel, for quality control and to estimate average insert size. All PCR products were purified on a Beckman Biomek FX using Montage PCR 96 Cleanup kits (Millipore), eluted in 100 µL of water, evaporated overnight, and resuspended in 30 µL of 3X SSC spotting buffer. Resuspended cDNA inserts (1 µg/µL) and negative controls (3X SSC with no nucleic acid) were arrayed from 96-well microtiter plates onto CMT-Gaps II (Corning, NY) slides using the Affymetrix Arrayer 417. Each clone was spotted 6X per slide. Spotting conditions were maintained between 19–20°C and 50–60% relative humidity. Slides were post-processed by baking at 80°C for 3 h, followed by a 2 min incubation in 1% SDS, a 2 min incubation in 95°C purified water, and 20 plunges into 100% ethanol at −20°C. After air-drying via centrifugation at 500 RPM for 5 min, all slides were stored in a vacuum-sealed, light-tight desiccator until hybridization.

For hybridization to the macroarray, each RNA sample derived from pools of *An. gambiae* or *An. funestus* representing multiple developmental stages and both sexes: first and third instar larvae, early and late pupae, and male and female adults. Prior to total RNA extraction, pools of 30 *An. funestus* or *An. gambiae* consisting of five individuals from each of the six stages/sexes, were prepared. From each pool (hereafter, sample), total RNA was extracted using Trizol. Following RNA extraction, samples were treated with 1.0 µL DNase I (Life Science Technology), extracted again with Trizol, and inspected by 1% agarose gel electrophoresis. First strand synthesis and labeling were performed using the Genisphere 3DNA Array 50 kit and Cy3 and Cy5 dyes as recommended (Genisphere), with 15 µg of total RNA from each sample. The Cy3 and Cy5 labeled cDNAs were combined and concentrated with Microcon microconcentrators (Amicon) prior to hybridization and washing as recommended by Genisphere. Of the seven hybridizations comprising the macroarray experiment, six consisted of *An. funestus* cDNA labeled with Cy3 and *An. gambiae* cDNA labeled with Cy5; these six hybridizations represented three biological and two technical replicates (3 biological×2 technical replicates = 6 slides). The seventh hybridization consisted of a Cy3-Cy5 dye swap to correct for potential dye bias.

Macroarray slides were scanned successively at two wavelengths corresponding to the absorption maximum of the Cy3 and Cy5 fluorochromes, 532 and 635 nm, using the Affymetrix 428 Array Scanner. Raw signal intensities were acquired based on predefined and manually aligned grids (48 columns×16 rows) using the adaptive circle algorithm in Jaguar 2.0 (Affymetrix, CA). Data were normalized by Loess curve in Genespring 5.1 (Silicon Genetics, CA) and filtered to retain only those elements with signal intensities >300 pixels in at least one channel.

## Results and Discussion

### cDNA library characteristics

**Figure 1:**
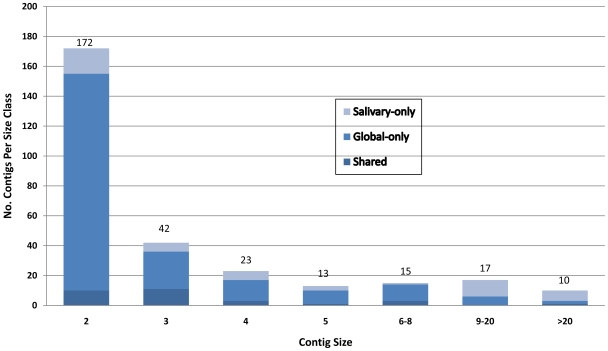
Histogram of 1,035 *An. funestus* EST contigs (clusters of ≥2 sequences) resulting from a combined assembly of ESTs from two sources. Sources were the whole body transcriptome of mixed developmental stages and sexes, and the adult female salivary glands. Numbers above the bars represent the total number of contigs per contig size class. Not shown are the 744 singletons containing only one EST.

A directionally cloned cDNA library was constructed from mixed stage F1 progeny (larvae, pupae, and adults) of field-collected *An. funestus* from Niono, Mali. Based on agarose gel electrophoresis of a random subset of inserts amplified by PCR (N = 435), average cDNA length was estimated at 1083±493 bp, with minimum and maximum lengths from ∼200 bp to ∼3000 bp. Sequencing of 3264 cDNAs from the 5′ end yielded 3020 sequence reads, of which ∼34% (1019) shared no significant similarity to mitochondrial or bacterial genomes, and met a 70-bp length threshold after end-trimming and vector screening. When clustered by themselves, these 1019 ESTs were assembled into 660 clusters of 135 contigs (≥2 sequences) and 525 singletons, with an overall average length of 493 bp. To take advantage of all available EST data from *An. funestus*, we re-clustered together with a previously characterized set of ESTs derived from *An. funestus* adult female salivary glands [8]. The average length of assembled ESTs was comparable (491 bp), but the total number of clusters increased to 1035, of which 292 were contigs of more than one sequence. A histogram of these 292 contigs from the combined assembly is given in Figure 1. The vast majority of assembled ESTs were represented by singletons or contigs containing only two sequences. A relatively small fraction of contigs were assembled from ESTs derived from both salivary glands and mixed developmental stages. As expected given low complexity and high abundance of sialome contents [8], functional annotation of these 1,035 transcripts indicated that salivary gland ESTs predominated in the largest contigs, especially those containing >20 sequences (Table 1 and Supplemental File S1, found at http://exon.niaid.nih.gov/transcriptome/A_funestus/S1/Af-S1-web.xls).

### Transcriptome conservation between An. funestus and An. gambiae

**Figure 2:**
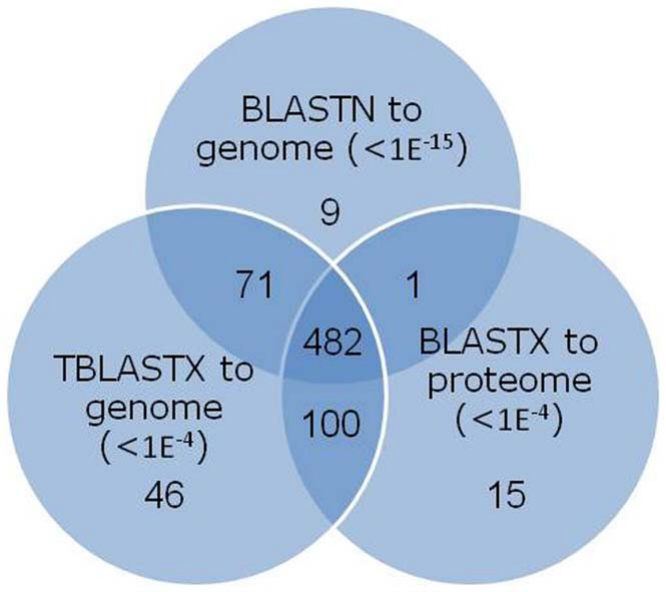
Venn diagram indicating the number of assembled *An. funestus* ESTs that showed significant similarity to the *An. gambiae* genome or proteome by one or more BLAST tools.

Maximum likelihood estimates of divergence time between *An. funestus* and *An. gambiae* range from 20–80 Mya [7]. As a first step in assessing sequence conservation between transcribed sequences in the two genomes, assembled *An. funestus* ESTs (contigs and singletons) were used to query the *An. gambiae* genome by three approaches: BLASTN and TBLASTX against the AgamP3 genome (using E-value cutoffs of <1×10^−15^ and <1×10^−4^, respectively), and BLASTX against the predicted AgamP3.4 proteins (E<1×10^−4^). (Inference of translation products from ESTs is described in section 3.3, below). The results are given as a Venn diagram in Figure 2. Depending upon the BLAST tool, 54–68% (563–700) of *An. funestus* assembled sequences shared significant sequence identity with the *An. gambiae* genome, most of these (482) by all three tools. BLASTN was least efficient compared to BLASTX and TBLASTX, probably owing in part to intron-exon structure in the genome and to lower levels of sequence conservation in untranslated (UTR) regions of transcripts. TBLASTX revealed more homologous amino acid sequences than BLASTX, possibly reflecting deficiencies in the predicted protein set of *An. gambiae*. The large number of assembled sequences that did not share significant sequence similarity with *An. gambiae* includes 278 sequences that did not match sequences in any database searched using BLASTX, including NR. These may represent novel gene products, but more likely, artifactual sequences and deficiencies in the ESTs: lack of an ORF due to 5′-truncation, frameshift errors, or short sequence.

Comparing available ESTs from both species by TBLASTX, 358 gene pairs were classified as homologous, of which 244 were considered orthologous (see Materials and Methods). Based on the location of genes in the *An. gambiae* reference genome, these orthologous pairs were distributed uniformly across the five chromosome arms. After manual alignment and filtering based on a minimum length of 50 aligned amino acids, 213 pairs remained. The mean length of aligned gene sequences was 130 amino acids (SD = 42; range = 50–259). The nucleotide and amino acid percent identity of orthologous pairs was 83.1% (SD = 7.4%) and 88.1% (SD = 10.3%), respectively. Interestingly, codon bias was significantly higher in the orthologs from *An. gambiae* than those from *An. funestus* according to two metrics, the codon bias index (CBI) and the effective number of codons (ENC). Higher codon bias in *An. gambiae* was associated with significantly higher G+C content at the third codon position (GC3) in the gene set from this species (mean CBI, ENC and GC3 for *An. funestus* were 0.49, 48 and 0.60; corresponding values for *An. gambiae* were 0.58, 41 and 0.72; *P*≪0.001 by t-test for all comparisons).

Previous studies using glass cDNA macroarrays have shown that detection of transcript presence primarily depends upon ≥80% nucleotide identity over at least 100 bp measured by raw BLASTN results [20]. We constructed *An. funestus* macroarrays by spotting randomly chosen PCR-amplified cDNA inserts onto glass slides. Slides were hybridized with labeled cDNA prepared from mixed developmental stages of *An. funestus* and *An. gambiae*. Because variation in signal intensity of hybridized cDNA could be due to sequence divergence and/or different expression levels between samples, no attempt was made to interpret the results in a quantitative fashion. Instead, a qualitative approach was taken. Based on signal intensities exceeding a threshold of 300 pixels in the Cy3 or Cy5 channel, transcripts were defined as “present” or “absent”. The 492 features analyzed on the array represented 429 unique contigs and singletons. Of these 429 transcripts, 265 (62%) were called “present” in *An. funestus*. Notably, of the 265 transcripts “present” in *An. funestus* samples, 248 (94%) transcripts also were deemed “present” *An. gambiae* samples. Thus, the vast majority of *An. funestus* transcripts were detected in the transcriptome of *An. gambiae*. These results suggest that the high rate of chromosomal rearrangement since the divergence of *An. gambiae* and *An. funestus* [21] has not been accompanied by a correspondingly high rate of genome-wide transcriptome evolution, although this generalization does not rule out accelerated evolution of particular gene classes.

### Functional annotation and proteome conservation

Any apparent insertions or deletions were manually removed from the assembled EST clusters containing multiple sequences. Based on three frame translations of all 1036 clusters, inferred complete or partial protein products were extracted from the largest ORF and used to query proteins in the NCBI NR database with the BLASTX tool. Slightly more than half of *An. funestus* clusters, 59% (607 of 1035), matched an entry in the NR protein database with an E-value below the cut-off (<1×10^−4^). Among those 607 clusters, ∼90% (550) shared greatest amino acid similarity within genus *Anopheles*; another ∼6% (35) were most similar to culicine mosquito proteins, mainly *Ae. aegypti*. Only ∼4% (22) were more similar to other, non-mosquito species.

In addition to comparisons with the NR protein database, these conceptual translation products were also compared to GO and protein motif databases using RPS-BLAST, in support of manual annotation. Upon joint inspection of the results of similarity searches of NR, GO and motif databases, EST contigs were assigned putative functions, and classified into broad functional categories, summarized in Table 1. The electronic version of the complete annotated catalog (Microsoft Excel format) with hyperlinks to web-based databases and to BLAST results are available as Supplemental File S2 and can be downloaded from http://exon.niaid.nih.gov/transcriptome/A_funestus/S2/Af-S2-web.xls. Fully 40% of the translation products failed to match other proteins in the databases, probably owing largely to lack of significant ORFs in ESTs comprised mainly of untranslated sequences. However, it is notable that ∼13% of the putative proteins, though they matched other proteins in the databases, had no known function, being part of the post-genomic mystery of “conserved hypothetical” proteins [22]. The remaining set of proteins whose function could be inferred span a very broad range of biological functions, underscoring the complexity of this set of *An. funestus* ESTs and their protein products.

**Figure 3:**
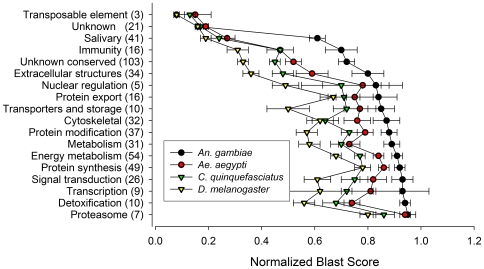
Average normalized BLAST scores (±SE) for protein comparisons between *An. funestus* and four other species. Black circle, *An. gambiae*; red circle, *Ae. aegypti*; green triangle, *Cx. quinquefasciatus*; yellow triangle, *D. melanogaster*). Corresponding functional categories (and number of protein comparisons in each) are indicated at left.

More detailed analysis was based on a subset of 506 “high quality” translation products whose raw self-BLAST scores exceeded 60 (see Materials and Methods). The proteome resulting from this conceptual translation of the *An. funestus* EST database was compared in a pairwise fashion with the proteomes of other mosquitoes (*An. gambiae*, *Ae. aegypti* and *Cx. quinquefasciatus*) and more distant relatives (*D. melanogaster*, *C. elegans, Arabidopsis thaliana* and *S. cerevisiae*). Comparison was based on normalized BLAST scores [referred to as the BLAST score ratio; 16] within the functional categories given in Table 1. The results provide insights about the patterns of protein evolution between taxonomic groups. As expected, conservation was highest between the pair of anopheline species and decreased with increasing phylogenetic distance (Figure 3, Table 2). This held true regardless of functional categorization. In addition, differences between taxonomic groupings were quite discrete. For example, there was relatively little overlap between mean scores (and their standard errors) for anopheline comparisons versus those derived from culicine comparisons (Figure 3), likely reflecting the deep ∼150 MY divergence between culicines and anophelines. Similarly, there was little overlap between mosquito scores and those derived from mosquito-Drosophila comparisons, representing an even deeper divergence of ∼260 MY.

**Table 2:** Similarity of Anopheles funestus proteins to best matching homologues of An. gambiae, Aedes aegypti, Culex quinquefasciatus, Drosophila melanogaster, Caenorhabditis elegans, Arabidopsis thaliana and Saccharomyces cerevisae.

		*An. gambiae*	*Ae. aegypti*	*C. quinquefasciatus*	*D. melanogaster*	*C. elegans*	*A. thaliana*	*S. cerevisae*
Functional Class	N	Mean (SE)	Mean (SE)	Mean (SE)	Mean (SE)	Mean (SE)	Mean (SE)	Mean (SE)
Proteasome	7	0.95(0.03)	0.94 (0.02)	0.86 (0.04)	0.80(0.03)	0.54 (0.06)	0.51 (0.05)	0.41 (0.06)
Detoxification	10	0.94(0.02)	0.74 (0.03)	0.68 (0.05)	0.56 (0.04)	0.26 (0.05)	0.25 (0.04)	0.19 (0.04)
Transcription	9	0.93 (0.10)	0.81 (0.11)	0.72 (0.10)	0.62 (0.12)	0.24 (0.05)	0.28 (0.07)	0.22 (0.04)
Signal transduction	26	0.93 (0.04)	0.82 (0.05)	0.75 (0.05)	0.61 (0.05)	0.36 (0.05)	0.27 (0.04)	0.25 (0.04)
Protein synthesis	49	0.92 (0.02)	0.86 (0.02)	0.78 (0.03)	0.78 (0.03)	0.57 (0.03)	0.56 (0.03)	0.52 (0.03)
Energy metabolism	54	0.91 (0.02)	0.84 (0.02)	0.77 (0.02)	0.68 (0.02)	0.40 (0.03)	0.29 (0.02)	0.26 (0.02)
Metabolism	31	0.89 (0.03)	0.73 (0.04)	0.70 (0.04)	0.58 (0.04)	0.36 (0.03)	0.24 (0.03)	0.21 (0.03)
Protein modification	37	0.88 (0.03)	0.79 (0.03)	0.73 (0.03)	0.57 (0.04)	0.35 (0.03)	0.28 (0.03)	0.23 (0.03)
Cytoskeletal	32	0.87 (0.05)	0.76 (0.05)	0.64 (0.05)	0.62 (0.05)	0.41 (0.06)	0.33 (0.05)	0.32 (0.05)
Transporters and storage	10	0.85 (0.05)	0.77 (0.06)	0.72 (0.05)	0.50 (0.08)	0.21 (0.07)	0.19 (0.06)	0.13 (0.03)
Protein export	16	0.84 (0.07)	0.75 (0.07)	0.71 (0.06)	0.67 (0.05)	0.44 (0.05)	0.32 (0.05)	0.24 (0.04)
Nuclear regulation	5	0.83 (0.05)	0.78 (0.15)	0.70 (0.15)	0.49 (0.05)	0.20 (0.05)	0.21 (0.04)	0.20 (0.04)
Extracellular structures	34	0.80 (0.06)	0.59 (0.06)	0.48 (0.04)	0.36 (0.03)	0.16 (0.01)	0.16 (0.01)	0.15 (0.01)
Unknown conserved	103	0.72 (0.03)	0.52 (0.03)	0.45 (0.02)	0.33 (0.02)	0.18 (0.01)	0.15 (0.01)	0.13 (0.01)
Immunity	16	0.70 (0.06)	0.47 (0.05)	0.47 (0.05)	0.31 (0.04)	0.20 (0.02)	0.16 (0.02)	0.15 (0.02)
Salivary	41	0.61 (0.03)	0.27 (0.03)	0.24 (0.03)	0.19 (0.02)	0.13 (0.01)	0.13 (0.01)	0.12 (0.01)
Unknown	21	0.16 (0.01)	0.19 (0.01)	0.17 (0.01)	0.16 (0.01)	0.17 (0.01)	0.16 (0.01)	0.15 (0.01)
Transposable element	3	0.08 (0.00)	0.15 (0.06)	0.13 (0.02)	0.08 (0.01)	0.11 (0.03)	0.07 (0.010	0.07 (0.010)

N, number of *An. funestus* proteins compared per functional class; Mean, average normalized blast scores; SE, standard error of the mean.

Comparative genomics also revealed different patterns of evolution based on functional categorization. The data in Table 2 suggest that the mean similarity scores differ between functional categories. Indeed, differences in the median values among categories with more than 15 sequences are greater than would be expected by chance (Kruskal-Wallis one way analysis of variance on ranks, based on *An. gambiae* comparison; *H* = 141.191, 11 df, *P*<0.001). The two categories with the highest mean scores, or highest amino acid sequence conservation, are responsible for protein synthesis and degradation (the proteasome), essential housekeeping functions. Of particular interest at the other end of the spectrum are the four categories whose mean similarity scores are strikingly lower than the rest, suggesting accelerated rates of evolution. In addition to the “conserved hypothetical” proteins of unknown function, the least conserved categories include adult saliva, immunity and extracellular structures (all with *P*<0.05 when compared to the protein synthesis category by Dunn's Multiple Comparison Method). Salivary, immunity and extracellular structural proteins all play important roles in how mosquitoes adapt to their nutritional and external environments and therefore could be of interest in disease control. Previous work has suggested accelerated evolution of genes relevant to hematophagy [23]. Most of the 60–100 secreted proteins in adult saliva have no known function, but are presumed to affect vertebrate hemostasis and inflammation, as well as assisting with sugar digestion and protecting blood and sugar meals from microbial growth [24]. Similarly, genes involved in immunity are also known to evolve rapidly, presumably in response to selective pressures imposed by pathogens and parasites [25], [26]. Included in this category are 17 immune genes, including antimicrobial peptides (defensin, cecropin, gambicin) and pattern recognition receptors (a peptidoglycan recognition protein and C-type lectin). In a previous study, we observed that the most highly adaptive *An. funestus* genes (such as genes coding for immune proteins) often differed most when compared to their *An. gambiae* orthologs [21]; the present analysis extends those findings. The extracellular structures category includes proteins (34) involved in peritrophic matrix synthesis (peritrophins) and cuticle formation (cuticle proteins), structures which function as protective barriers against complex, challenging and changing environments. Recent unexpected findings from adult Colorado potato beetles revealed that cuticular protein genes were highly induced by exposure to an insecticide as well as by dry environmental conditions 2–3 weeks after adult moulting, suggesting that the insect can increase cuticular component deposition at the adult stage in response to environmental stresses [27]. Thus, the relatively rapid evolution of genes in these three functional categories– salivary, immunity and extracellular structures—may be driven by similar types of environmental stresses. In this light, the fact that proteins in the “unknown function” category seem to be evolving at similarly accelerated rates as salivary, immunity and extracellular structural proteins is suggestive that they may also be responding to environmental factors and could be unrecognized members of these three functional classes.

It has been reported previously that exogenous detoxification genes are under a fast pace of evolution [28], in particular the set comprised by glutathione transferases (GST), cytochrome P450 (P450) and carboxylesterases, where large family expansions have been observed in mosquitoes [29], as opposed to the honey bee [30], for example. In contrast, our results (Table 2) showed relative conservation of this category. The apparent contradiction can be accounted for by the limited size of our set (only 10 products), and more importantly by the fact that this set contains– and is dominated by—seven conserved endogenous detoxifiers including short chain dehydrogenases and catalase, as well as dopamine acetyl transferase. The set contained only one P450 and two GST products representing typical exogenous detoxifiers associated with rapid evolutionary rates. For comparison, the current *Ae. aegypti* proteome lists 178 proteins with the KOG domain of cytochrome P450, averaging a stardardized score of 0.652 and 0.529 against their best matches to the *Cx. quinquefasciatus* and *An. gambiae* proteomes, respectively, values that would place these genes at as fast a pace of evolution as immunity genes shown in our Table 2 analysis.

### Concluding remarks

Of all mosquitoes in Family Culicidae, only anopheline mosquitoes are capable of transmitting human malaria, for reasons that remain obscure. At present, *An. gambiae* is the only completely sequenced genome of any anopheline species. Novel approaches to fighting malaria may reveal themselves in the effort to understand the genetic, behavioral and physiological differences in vector ability among anopheline species as well as the absolute block to malaria vector ability that differentiates culicines and anophelines. Toward that effort, we have provided an initial catalog of ∼1000 non-redundant transcripts that will facilitate the development of gene models for the anticipated whole genome sequencing of *An. funestus* and additional anopheline species. Our comparative genomic analysis revealed that conserved hypothetical proteins of unknown function are evolving at accelerated rates similar to genes involved in hematophagy, immunity and formation of extracellular structures, emphasizing that functional characterization will benefit from a database of diverse anopheline species. Given the very rapid pace of technological developments, future efforts to characterize anopheline transcriptomes should be greatly aided by high-throughput methods such as RNA sequencing [31].

## Supporting Information

File S1 Excel spreadsheet including detailed functional annotation of A. funestus EST transcripts
(11.23 MB XLS)

File S2 Excel file containing functional annotation of A. funestus ESTs based on their conceptual translation products
(2.95 MB XLS)
